# Supplementary material for: Cortistatin as a Novel Multimodal Therapy for the Treatment of Parkinson’s Disease
Source: Int J Mol Sci. 2024 Jan 5;25(2):694. doi: 10.3390/ijms25020694 (PMC10815070; doi:10.3390/ijms25020694)
Supplement: Supplementary file 1 [file ijms-25-00694-s001.zip › Serrano-Martínez et al-IJMS-Supplementary- final-REV2.pdf]

SUPPLEMENTARY MATERIALS

Cortistatin as a novel multimodal therapy for the treatment of Parkinson´s Disease

Ignacio Serrano-Martínez[1,†], Marta Pedreño[1,†], Julia Castillo-González[1], Viviane Ferraz-de-Paula[1], Pablo Vargas-Rodríguez[1], Irene Forte-Lago[1], Marta Caro[1], Jenny Campos-Salinas[1], Javier Villadiego[2,3,4], Pablo Peñalver[5], Juan Carlos Morales[5], Mario Delgado[1], and Elena González-Rey[1,*]

[1]Department of Cell Biology and Immunology, Institute of Parasitology and Biomedicine Lopez-Neyra (IPBLN), CSIC, PT Salud, 18016 Granada, Spain; ignacioserrano23@ipb.csic.es (I.S.-M.); marta.pedreno@outlook.es (M.P.); juliacastillo@ipb.csic.es (J.C.-G.); Ferraz.vi@gmail.com (V.F.-de-P.); Pablo.vargas@ipb.csic.es (P.V.-R.); irene.forte@ipb.csic.es (I.F.-L.); martacm@ipb.csic.es (M.C.); jenny@ipb.csic.es (J.C.-S.); mdelgado@ipb.csic.es (M.D.)

[2]Instituto de Biomedicina de Sevilla (IBiS), Hospital Universitario Virgen del Rocío/CSIC/Universidad de Sevilla, 41013 Sevilla, Spain; fvilladiego@us.es (J.V.)

[3]Departamento de Fisiología Médica y Biofísica, Facultad de Medicina, Universidad de Sevilla, 41009 Sevilla, Spain

[4]Centro de Investigación Biomédica en Red sobre Enfermedades Neurodegenerativas (CIBERNED), 28029 Madrid, Spain

[5]Department of Biochemistry and Molecular Pharmacology, Institute of Parasitology and Biomedicine Lopez-Neyra (IPBLN), CSIC, PT Salud, 18016 Granada, Spain; pablo@ipb.csic.es (P.P.); jcmorales@ipb.csic.es (J.C.M.)

***Corresponding author**: Elena González-Rey, Institute of parasitology and Biomedicine Lopez-Neyra (IPBLN-CSIC), PT Salud, Granada, Spain. Email: elena.g@csic.es. Tfn: 34 958181670; Fax: 34 958181633; ORCID: 0000-0003-3917-9020

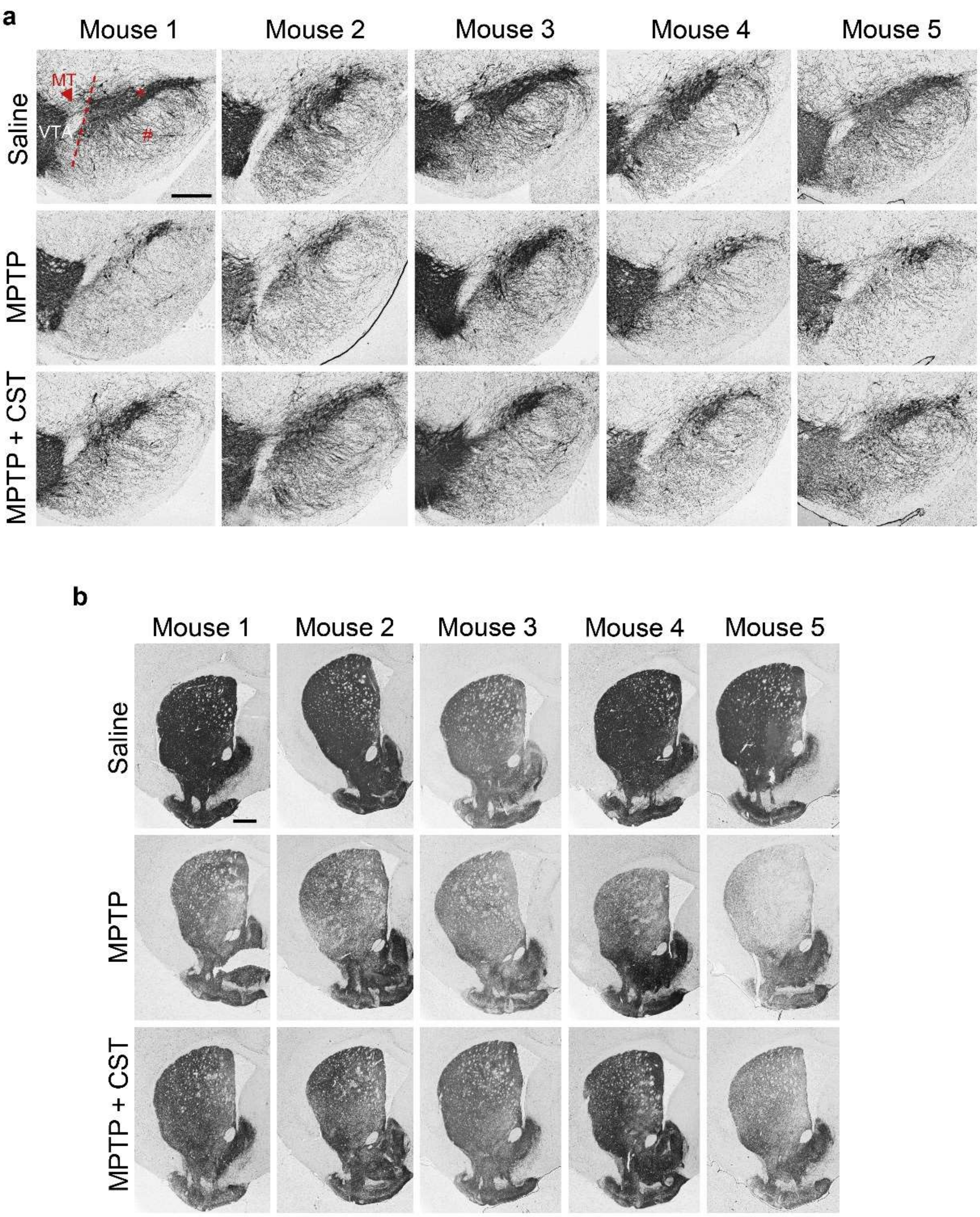

Supplementary Figure S1. Cortistatin protects against dopaminergic-related toxicity induced by MPTP. C57BL/6 mice were administrated with MPTP (20 mg/kg; 4 i.p. injections at 2 h intervals), and then treated with vehicle (MPTP group) or with cortistatin (MPTP+CST group) for 7 consecutive days, beginning 2 h after the last MPTP injection. Animals i.p injected with saline instead of MPTP were used as controls (saline group). After this, brains were obtained and dissected as described in Methods section. Representative images of the tyrosine hydroxylase (TH, depicted in black staining) immunoreactivity showing neuron bodies (indicated by red asterisk) and projections (indicated by red hash) in the SNpc (**a**), and neuronal fibers in the striatum (**b**) for each mouse. Scale bar: 500 µm. n = 5 sections/mouse; N= 5 mice/group. SNpc: substantia nigra pars compacta; VTA: ventral tegmental area; MT: medial terminal nucleus.

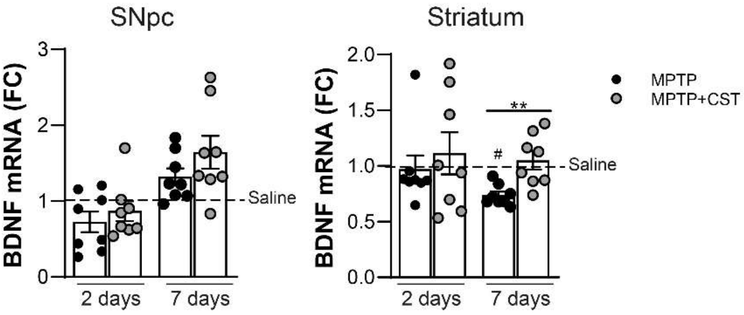

Supplementary Figure S2. Cortistatin preserves *Bdnf* expression in the acute model of MPTP. Experimental PD was induced in mice and treated with cortistatin as indicated in Supplementary Figure 1. Midbrains were isolated 2 or 7-days post MPTP and dissected as described in the Methods section. *Bdnf* gene expression levels in the SNpc (left) and the striatum (right) were quantified by real-time qPCR analyses and normalized to *Gapdh* expression. Data was represented as the fold change of the mRNA levels in each brain tissue isolated from saline-treated control mice (set at 1; dashed line). N = 8 mice/group. All data are reported as mean ± s.e.m. Each dot represents individual mice. * $p<0.05$, ** $p<0.01$, *** $p<0.001$, vs MPTP-mice; # $p<0.05$, ## $p<0.01$, #### $p<0.0001$, vs saline-treated mice.

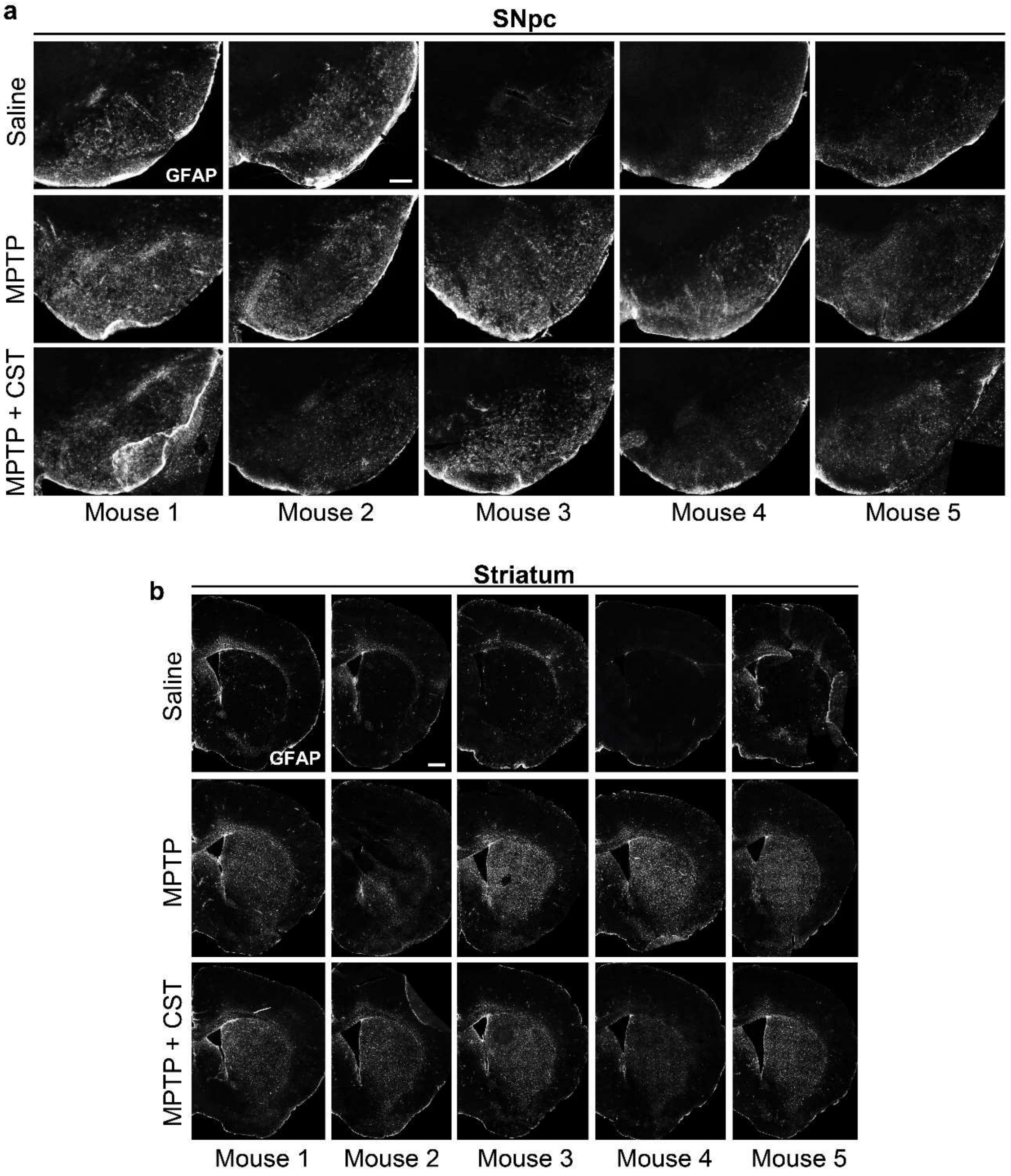

Supplementary Figure S3. Treatment with cortistatin reduces MPTP-induced nigral astrogliosis. Experimental PD was induced in mice and treated with cortistatin as indicated in Supplementary Figure 1. Brains were isolated seven days post-MPTP injection and coronal sections corresponding to the SNpc-containing ventral mesencephalic (**a**) and striatum (**b**) regions were immunostained for GFAP (white staining). Representative images of the GFAP immunoreactivity showing activated astrocytes in the SNpc (**a**, scale bar: 200 µm) and striatum (**b**, scale bar: 500 µm) for each mouse. N= 5 mice/group. GFAP: Glial fibrillary acidic protein, SNpc: substantia nigra pars compacta.

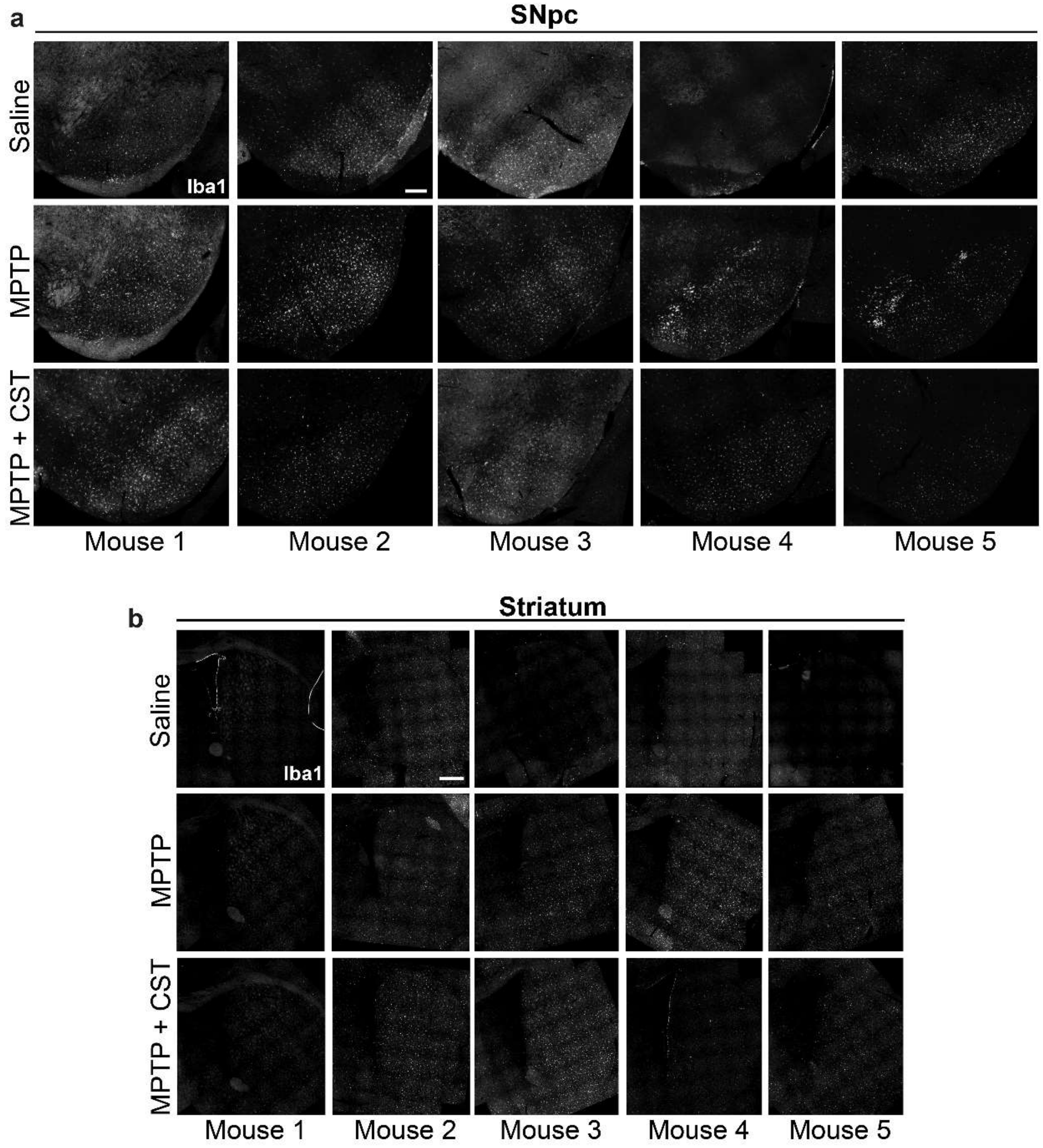

Supplementary Figure S4. Cortistatin reduces MPTP-induced reactive microglia in the SNpc. Experimental PD was induced in mice and treated with cortistatin as indicated in Supplementary Figure 1. Brains were isolated seven days post-MPTP injection and coronal sections corresponding to the SNpc-containing ventral mesencephalic (**a**) and striatum (**b**) regions were immunostained for Iba1 (white staining). Representative images of the Iba1 immunostaining showing reactive microglia in the SNpc (**a**, scale bar: 200 µm) and striatum (**b**, scale bar: 500 µm) for each mouse. N= 5 mice/group. Iba1: Ionized calcium binding adaptor molecule 1, SNpc: substantia nigra pars compacta.

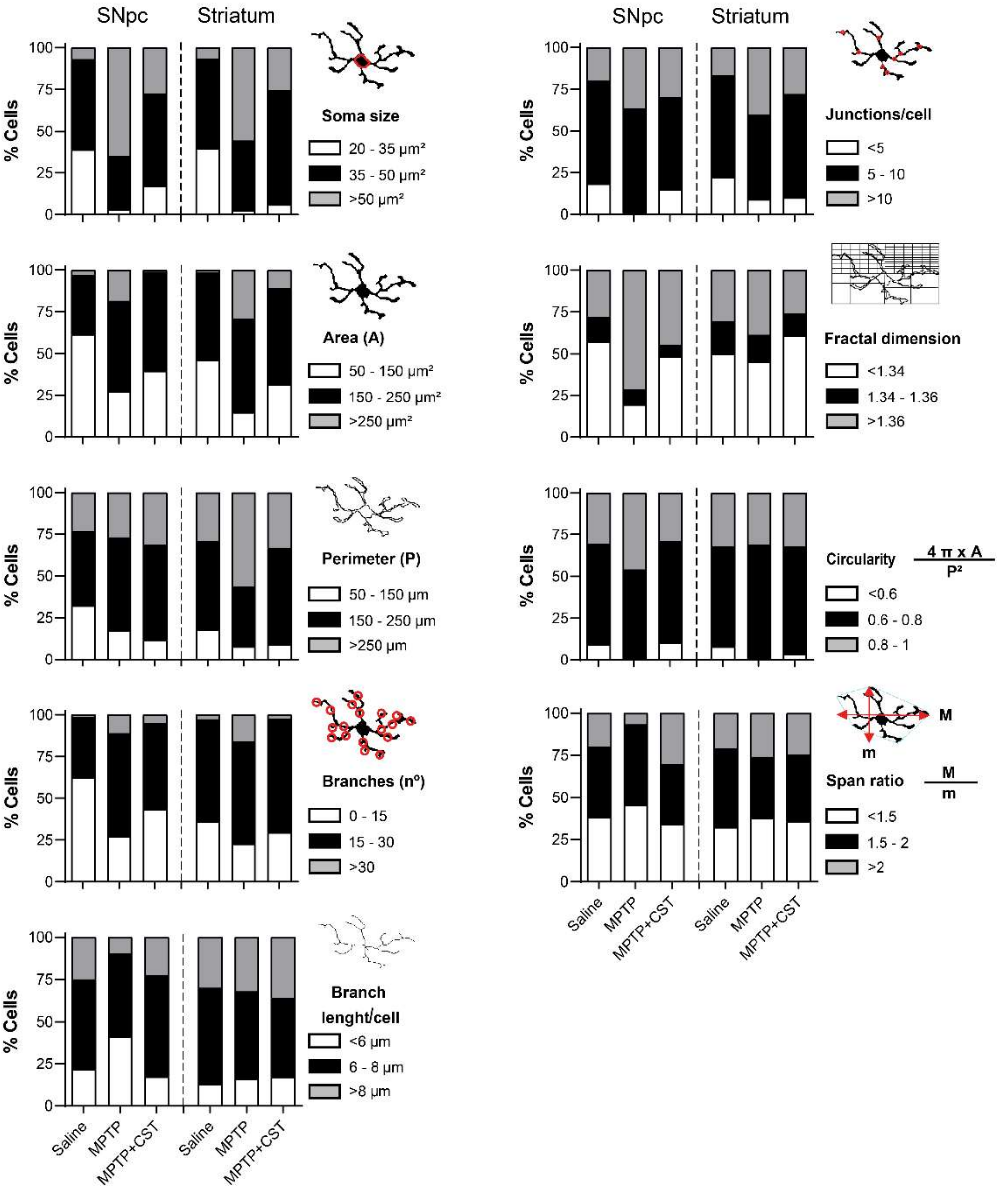

Supplementary Figure S5. Cortistatin administration modulates microglia morphotypes in brain regions affected by MPTP. Experimental PD was induced in mice and treated with cortistatin as indicated in Supplementary Figure 1. Brains were isolated seven days post-MPTP injection and coronal sections corresponding to the SNpc-containing ventral mesencephalic and striatum regions were immunostained for Iba1. The Iba-1+ microglial morphotypes in SNpc and striatum from brains isolated from saline, MPTP and MPTP+CST mice were characterized following different cell shape descriptors. Each of these morphological traits were distributed in 3 groups: soma size (20-35, 35-50, >50µm^2); area (50-150, 150-250, >250µm^2); perimeter (50-150, 150-250, >250µm); number of branches (0-15, 15-30, >30µm^2); branch length/cell (<6, 6-8, >8µm); number of cell junctions/cell (<5, 5-10, >10); fractal dimension (arbitrary units-a.u; <1.34, 1.34-1.36, >1.36); circularity (a.u; <0.6, 0.6-0.8, 0.8-1); and span ratio (a.u; <1.5, 1.5-2, >2). Graphs represent the percentage of cells in each range for each descriptor. n = 65-75 cells/saline, n = 75-90 cells/MPTP, n = 70-85 cells/MPTP+CST (for both SNpc and striatum) were analyzed; N= 5 mice/group. Iba1: Ionized calcium binding adaptor molecule 1, SNpc: substantia nigra pars compacta.

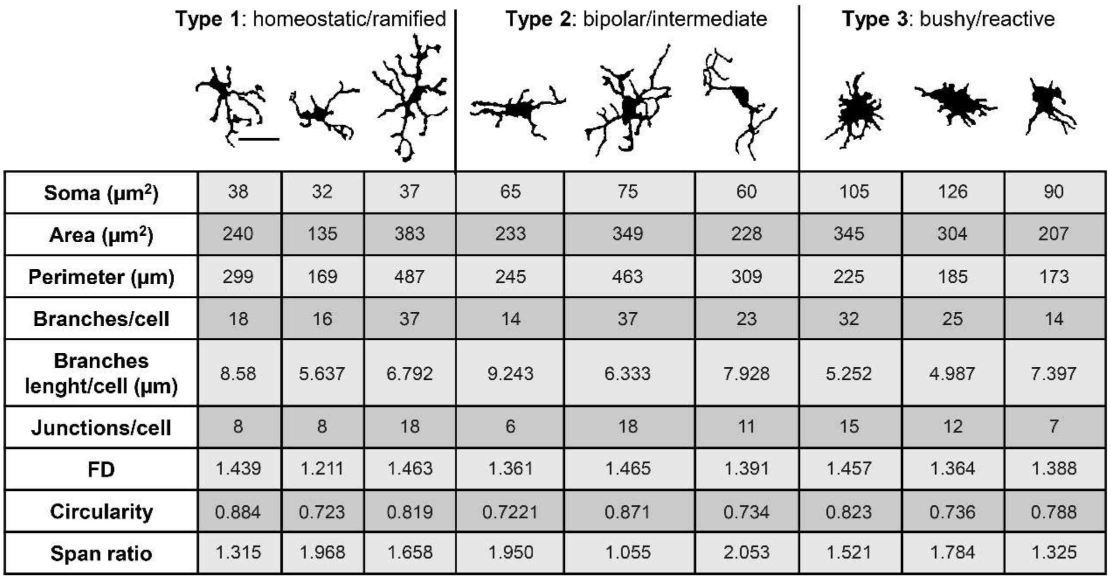

	**Type 1**: homeostatic/ramified			**Type 2**: bipolar/intermediate			**Type 3**: bushy/reactive		
**Soma (µm²)**	38	32	37	65	75	60	105	126	90
**Area (µm²)**	240	135	383	233	349	228	345	304	207
**Perimeter (µm)**	299	169	487	245	463	309	225	185	173
**Branches/cell**	18	16	37	14	37	23	32	25	14
**Branches lenght/cell (µm)**	8.58	5.637	6.792	9.243	6.333	7.928	5.252	4.987	7.397
**Junctions/cell**	8	8	18	6	18	11	15	12	7
**FD**	1.439	1.211	1.463	1.361	1.465	1.391	1.457	1.364	1.388
**Circularity**	0.884	0.723	0.819	0.7221	0.871	0.734	0.823	0.736	0.788
**Span ratio**	1.315	1.968	1.658	1.950	1.055	2.053	1.521	1.784	1.325

Supplementary Figure S6. Schematic representation of microglia-specific morphological prints. A visual representation is provided to depict distinct morphological characteristics specific to microglia identify in this study. Binary images, exemplifying morphological distinctions in Iba1+ cells, were chosen from mice treated with saline (type 1), MPTP (type 3), and cortistatin-treated mice (MPTP+CST; type 2), irrespective of the brain region. Scale bar: 20 µm. The table compiles a set of descriptors essential for identifying microglia morphotypes. These descriptors encompass the analysis of cell shape (soma size, area, and perimeter), the cellular network of projections (evaluation of the skeleton, including the number and length of branches and junctions), and cellular complexity (fractal dimension-FD, circularity, and span ratio).

Gene	Primer sequence (5'-3')
Gapdh (NM_001411843.1)	Fw: AACTTTGGCATTGTGGAAGG<br>Rv: ACACATTGGGGGTAGGAACA
Th (NM_009377.2)	Fw: GGTATACGCCACGCTGAAGG<br>Rv: TAGCCACAGTACCGTTCCAGA
Dat (NM_010020.3)	Fw: CTTCTCCTCTGGCTTCGTTGT<br>Rv: CAGGGTAGATGATGAAGATCAACC
Bdnf (NM_001048141.1)	Fw: CCCTCCCCCTTTTAACTGAA<br>Rv: GCCTTCATGCAACCGAAGTA
TNF-α (NM_013693.3)	Fw: GCGACGTGGAACTGGCAGAAGAG<br>Rv: TGAGAGGGAGGCCATTTGGGAAC
IL1β (NM_008361.4)	Fw: CTCCATGAGCTTTGTACAAGG<br>Rv: TGCTGATGTACCAGTTGGGG

Table S1. Sequence of primers used for real-time PCR quantifications. Rv: reverse; Fw: forward. *Gapdh*, glyceraldehyde-3-phosphate dehydrogenase; *Th,* tyroxine hydroxylase; *Dat,* dopamine transporter; *Bdnf*, brain-derived neurotrophic factor; *TNF-α*, tumor necrosis factor alpha; *IL1β*, interleukin 1 beta.
